# Combined Experimental and Computational Approaches for Ternary Solid Dispersions to Enhance the Oral Bioavailability of Penfluridol

**DOI:** 10.3390/pharmaceutics17121546

**Published:** 2025-11-30

**Authors:** Masoud Mamani, Gyu Lin Kim, Su Min Kil, Hyo-Kyung Han

**Affiliations:** College of Pharmacy, Dongguk University, Dongguk-ro-32, Ilsan-Donggu, Goyang 10326, Republic of Korea

**Keywords:** penfluridol, solid dispersion, random forest regression, poloxamer 407, PVP-K30

## Abstract

**Background:** Penfluridol is a long-acting oral antipsychotic used for the treatment of schizophrenia. Although the prolonged half-life of penfluridol allows once-weekly dosing, improving patient compliance, its therapeutic potential is limited by low aqueous solubility and poor oral absorption. This study aimed to enhance the dissolution and oral bioavailability of penfluridol using solid dispersion technology. **Methods:** Ternary solid dispersions of penfluridol were prepared using a solvent evaporation method with various hydrophilic carriers. Following prescreening of polymeric carriers, the formulation composition was optimized using a random forest regression model. Structural characteristics and drug release behavior of the optimized formulation (PF-SD5) were evaluated through in vitro studies. Pharmacokinetic studies in rats were conducted to assess the effectiveness of PF-SD5 in enhancing oral bioavailability. **Results:** The optimized PF-SD5 formulation, comprising penfluridol, poloxamer 407, and polyvinylpyrrolidone K30 in a 1:3:1 ratio, exhibited a 117-fold increase in aqueous solubility compared with the pure drug. PF-SD5 achieved nearly complete drug release within 1 h across a pH range from acidic to neutral. Spectroscopic, microscopical, and thermal analyses confirmed that penfluridol transformed into an amorphous form and established molecular interactions within the carrier matrix. Pharmacokinetic studies in rats revealed approximately a 1.9-fold increase in oral bioavailability. **Conclusions:** Combining solid dispersion technology with machine learning-guided optimization provides an effective strategy for enhancing the oral absorption of poorly soluble penfluridol.

## 1. Introduction

Oral administration accounts for approximately 90% of commercially available pharmaceutical dosage forms [1]. Oral dosage forms are non-invasive, convenient, easy to use, and cost-effective, leading to greater patient adherence [2]. However, when an active pharmaceutical ingredient exhibits low aqueous solubility, oral formulations face substantial challenges, including poor bioavailability and suboptimal in vivo efficacy. Inadequate water solubility restricts drug dissolution and, consequently, oral bioavailability, particularly for Biopharmaceutics Classification System (BCS) Class II, such as penfluridol [3,4]. Penfluridol is a long-acting antipsychotic used for the treatment of chronic schizophrenia [5]. Unlike conventional oral antipsychotics, which require daily administration, penfluridol provides sustained therapeutic activity owing to its exceptionally long half-life of approximately 66 h in humans [6]. This property allows for once-weekly dosing, potentially improving patient adherence. The prolonged half-life and sustained activity of penfluridol are attributed to its high lipophilicity and extensive distribution into adipose tissues, where it forms a depot that gradually releases the drug [7]. Clinically, weekly administration of penfluridol achieves efficacy comparable to daily regimens of chlorpromazine without major adverse effects [8]. Despite these advantages, penfluridol exhibits poor aqueous solubility and limited oral bioavailability [9]. Several formulation strategies have been explored to overcome the solubility limitations of penfluridol, including self-microemulsifying drug delivery systems (SMEDDS) and molecular salt forms of penfluridol [10,11]. Although these approaches have shown promise, they also present certain drawbacks. Producing salt forms requires chemical modification, which may complicate manufacturing and affect stability. Additionally, introducing a new salt form late in development may necessitate repeating toxicology, formulation, and stability studies, increasing time and cost demands [12]. In contrast, SMEDDS formulations rely on high concentrations of surfactants, raising concerns regarding safety, cost, and handling [13,14]. Therefore, there is a need for new oral formulations of penfluridol that are cost-effective, stable, and scalable.

Solid dispersion (SD) technology, which involves dispersing pharmaceutical agents within a hydrophilic carrier matrix, has shown considerable potential for enhancing the dissolution rate and systemic absorption of BCS Class II compounds [15]. Improvements in dissolution are achieved through particle size reduction, increased wetting, and conversion of the drug to an amorphous form [16]. These effects collectively promote supersaturation upon dissolution, thereby enhancing bioavailability [17]. SD strategies offer distinct advantages, including formulation simplicity and broad applicability for enhancing the solubility of poorly water-soluble drug candidates [18]. Given the availability of polymers with diverse physicochemical and thermochemical properties, various polymer combinations can be employed in SD formulations to modulate the extent and rate of drug dissolution [19]. However, optimizing SD formulations through purely experimental approaches can be time-consuming and resource-intensive. Therefore, computational modeling and machine learning (ML) tools have emerged as powerful aids for guiding formulation design and optimization in recent years. Specifically, data-driven methods can be applied to predict formulation performance and potentially reduce the need for experimental evaluations [20]. Among ML algorithms, random forest regression (RFR) has demonstrated exceptional capability in modeling complex, nonlinear relationships between formulation variables and outcomes such as solubility or dissolution rate [21,22]. RFR operates by constructing an ensemble of decision trees and averaging their predictions, which reduces overfitting and enhances predictive reliability [23]. The value of RFR in pharmaceutical formulation research lies in its suitability for handling complex and limited datasets. The inherent ensemble-based learning mechanism of RFR contributes to highly reliable predictive outcomes, even under conditions of restricted experimental observations. Consequently, RFR is considered an appropriate methodology for optimizing formulation compositions [24,25]. Nevertheless, no prior study has applied computational or ML-assisted approaches to predict and optimize SD formulations of penfluridol. Furthermore, literature information on the formulations improving the bioavailability of penfluridol is extremely limited, with no prior work using SDs. Therefore, this study aimed to combine experimental and ML-assisted approaches to fabricate ternary SD formulations incorporating hydrophilic polymers, improve the dissolution and systemic exposure of penfluridol, and optimize their compositions. To our knowledge, this is the first study to combine experimental and ML-assisted approaches for SD formulations of penfluridol.

## 2. Materials and Methods

### 2.1. Materials

Penfluridol (C_28_H_27_ClF_5_NO, purity > 98.0%) was sourced from Tokyo Chemical Industry Co., Ltd. (Tokyo, Japan). Hydroxypropyl methyl cellulose (HPMC, mw: ~86,000 g/mol, CAS 9004-65-3), ammonium formate, and phosphoric acid were purchased from Sigma-Aldrich (St. Louis, MO, USA). The following polymers were provided by BASF (Ludwigshafen, Germany): poloxamer 407 (P407, mw: ~12,000 Da, CAS 9003-11-6), poloxamer 188 (P188, mw: ~8400 Da, CAS 9003-11-6), polyvinylpyrrolidone K12 (PVP-K12, mw: 2000~3000 g/mol, CAS 9003-39-8), polyvinylpyrrolidone K30 (PVP-K30, mw: 40,000~55,000 g/mol, CAS 9003-39-8), and Kollidon VA64 (Co-PVP, mw: 45,000~70,000 g/mol, CAS 25086-89-9). Simulated gastric fluid (SGF) and simulated intestinal fluid (SIF) were prepared according to US pharmacopeia. All other chemicals were of analytical grade, and the solvents conformed to high-performance liquid chromatography (HPLC)-grade standards.

### 2.2. Carrier Screening and SD Preparation

To identify suitable carriers, SDs were prepared using a solvent evaporation method with a drug-to-carrier ratio of 1:5. Solvent selection was based on the solubility characteristics of each carrier. HPMC was dissolved in 70% ethanol, whereas dichloromethane was used for the remaining carriers. Penfluridol and the respective carrier were dissolved in the appropriate solvent under continuous magnetic stirring at 300 rpm for 15 min at ambient temperature. The solvent was subsequently removed via rotary evaporation at 60 rpm and 40 °C, followed by vacuum drying. The dried products were milled and then passed through a 60-mesh screen (sieve opening size of 250 µm). Solubility enhancement of the prepared SDs was determined in distilled water to select the optimal carriers for further characterization.

Following initial screening, ternary SDs were fabricated using two selected carriers blended at various ratios (Table 1) to achieve the desired dissolution properties. Physical mixtures (PMs) were prepared separately by trituration using the geometric dilution technique to ensure homogeneous blending.

### 2.3. Solubility Studies

Each formulation, equivalent to 5 mg of penfluridol, was added to 1 mL of distilled water and stirred continuously at 100 rpm at 37 °C for 48 h to reach equilibrium. Samples were filtered using a 0.45 μm syringe filter, and the concentration of penfluridol in the filtrate was determined by HPLC assay.

### 2.4. In Vitro Drug Release Studies

The dissolution profiles of the SD formulations were assessed using the USP Apparatus II (paddle method). Each formulation (pure drug, PM, or SD; equivalent to 20 mg of penfluridol) was encapsulated in hard gelatin capsules. The dissolution medium consisted of 900 mL of phosphate buffer (pH 6.8) with 0.3% tween 80 and was maintained at 37 °C ± 0.5 °C, with the paddle rotating at a speed of 50 rpm. Samples were withdrawn at predetermined time intervals, filtered through a 0.45 μm syringe filter, and analyzed for penfluridol content using HPLC. Dissolution studies were also performed in SGF and SIF to reflect in vivo conditions.

### 2.5. Machine Learning

Drug release at 1 h was predicted using an RFR algorithm. The model was implemented in Python 3.10 using the scikit-learn library (version 1.1.3). The first set of dissolution data, including time, carrier ratios, and corresponding drug release, was used to train the RFR model. These features served as input variables, while the output variables represented the predicted percentage of drug release at 1 h. Model performance was evaluated using multiple metrics, yielding an R^2^ score of 0.92, a root mean squared error (RMSE) of 10.28, a mean absolute error (MAE) of 6.92, and a five-fold cross-validation score of 0.906. This approach leverages the strength of RFR to model complex, nonlinear patterns in pharmaceutical dissolution data.

### 2.6. Structural and Morphological Characterizations

Fourier transform infrared spectroscopy (FTIR) was employed to investigate potential molecular interactions between the drug and polymers within the SDs. Spectra were acquired using an ATR-FTIR spectrophotometer (Nicolet™ iS™ 5, Thermo Fisher Scientific, Waltham, MA, USA) over a wavenumber range of 4000–400 cm^−1^, with 64 co-added scans and a spectral resolution of 4 cm^−1^. Differential scanning calorimetry (DSC Q2000, TA Instruments, New Castle, DE, USA) was employed to examine the thermal behavior of the samples. Accurately weighed samples were sealed in standard aluminum pans and heated from 25 °C to 300 °C at a linear rate of 10 °C/min under a nitrogen purge (50 mL/min). The instrument was calibrated using high-purity indium. X-ray powder diffraction (XRPD) analysis was conducted at the Korea Basic Science Institute (Daegu, Republic of Korea) to assess drug crystallinity.

Surface morphology was examined using a scanning electron microscope (COXEM EM-30 plus, Daejeon, Republic of Korea). Samples were mounted on aluminum stubs using double-sided carbon tape and coated with a thin gold layer using a sputter coater to enhance electrical conductivity. SEM images were obtained under high vacuum conditions at 15 kV, with representative micrographs captured at multiple magnifications to assess particle shape and surface texture.

### 2.7. Pharmacokinetic Studies in Rats

Pharmacokinetic studies were conducted using male Sprague–Dawley rats in compliance with ARRIVE guidelines (https://arriveguidelines.org/ (accessed on 5 August 2025) and approved by the institutional review committee of Dongguk University (IACUC-2023-038-2). Rats (7 weeks old, 220–250 g, n = 12) were obtained from Orient Bio Co., Ltd. (Seongnam, Republic of Korea) and housed two per cage under controlled conditions (22–25 °C, 50% relative humidity, 12 h light/dark cycle), with free access to water and food. Rats were fasted for 12 h before dosing, with water allowed ad libitum. Animals were randomly assigned to two treatment groups (n = 6 per group). Each formulation (pure penfluridol or PF-SD5) was dispersed in 0.5% aqueous methylcellulose and administered via oral gavage at a dose equivalent to 10 mg/kg of penfluridol. Blood samples (0.3 mL) were collected at predetermined intervals, centrifuged, and plasma was stored at −80 °C until analysis by LC/MS/MS assay.

### 2.8. Analytical Methods

In vitro samples: The concentration of penfluridol was determined using an isocratic HPLC assay. Chromatographic separation was performed on a reversed-phase C18 column (Gemini C18, 4.6 × 150 mm, 5 μm; Phenomenex, Torrance, CA, USA). The mobile phase consisted of methanol and 0.2% triethylamine (70:30, *v*/*v*), with the pH adjusted to 2.5 using phosphoric acid. The flow rate was maintained at 0.4 mL/min, and the column temperature was set to 30 °C. UV detection was performed at 219 nm. A linear standard curve (r^2^ > 0.999) was constructed over the concentration range of 1–100 μg/mL.

In vivo samples: Plasma concentrations of penfluridol were analyzed using LC/MS/MS. Chromatographic separation was performed on a Kinetex C18 100 Å column (100 × 4.6 mm, 2.6 μm; Phenomenex, Torrance, CA, USA) with an isocratic mobile phase composed of acetonitrile and 5 mM ammonium formate (70:30, *v*/*v*) at a flow rate of 0.5 mL/min. The column temperature was maintained at 40 °C. Mass spectrometric detection was conducted using a TSQ Quantis Triple Quadrupole Mass Spectrometer (Thermo Fisher Scientific, Waltham, MA, USA) equipped with a heated electrospray ionization (H-ESI) source operating in positive ion mode. The precursor/product ion pairs (*m*/*z*) were 524.183/202.983 for penfluridol and 271.15/154.97 for tolbutamide (internal standard). Argon was used as the collision gas. Calibration standards were prepared over a concentration range of 5–500 ng/mL and demonstrated excellent linearity with a correlation coefficient (r^2^) of 0.999. Plasma samples (90 µL) were mixed with 270 µL of acetonitrile, vortexed for 10 min, and centrifuged at 22,250× *g* for 10 min at 4 °C. The resulting supernatant was filtered, and 90 µL of the filtrate was combined with 10 µL of the internal standard solution (5 µg/mL tolbutamide) before injection.

### 2.9. Pharmacokinetic and Statistical Analysis

Pharmacokinetic parameters were determined using noncompartmental analysis. Statistical analyses were performed using IBM SPSS Statistics (version 26, Armonk, NY, USA). Results are presented as mean ± standard deviation. Statistical comparisons were conducted using one-way analysis of variance (ANOVA), with significance set at *p* < 0.05.

## 3. Results and Discussion

### 3.1. Carrier Screening and Solubility Enhancement

**Figure 1:**
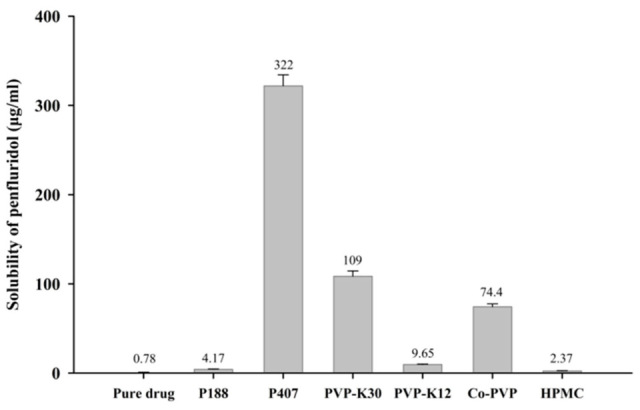
Effect of hydrophilic polymers on aqueous solubility of penfluridol in SD formulations (mean ± standard deviation [s.d.], n = 3).

SDs of penfluridol were prepared using various hydrophilic polymers, and their effectiveness in enhancing drug solubility is summarized in Figure 1. Among tested carriers, P407 produced the highest solubility enhancement (322 ± 12.3 µg/mL), followed by PVP-K30 (109 ± 5.89 µg/mL), compared with the pure drug (0.78 ± 0.02 µg/mL). These correspond to approximately 413-fold and 140-fold increases for P407 and PVP-K30, respectively. The superior solubility enhancement observed with P407 may be attributed to its non-ionic and hydrophilic nature, which lowers surface tension, improves wetting, and promotes micellar solubilization, thereby facilitating the aqueous dispersion and hydration of hydrophobic molecules [26]. Similarly, the enhanced solubility with PVP-K30 is due to its hydrophilic nature, which increases wettability and water penetration into the SD matrix. Moreover, PVP-K30 is known to inhibit drug recrystallization by restricting molecular mobility and forming hydrogen bonds with drug molecules [27,28]. These interactions stabilize the amorphous form of the drug, preventing crystallization and improving both physical stability and dissolution performance. Additionally, the inclusion of PVP-K30 mitigates the stickiness typically associated with poloxamer-based dispersions, enhancing powder flowability and processability. Therefore, a ternary SD system combining P407 and PVP-K30 was designed to leverage the solubilizing properties of P407 and the crystallization-inhibiting effects of PVP-K30 to maximize drug dissolution.

### 3.2. Formulation Optimization

**Figure 2:**
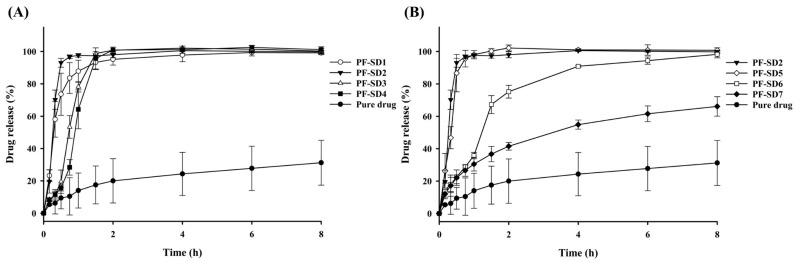
Dissolution profiles of SD formulations in phosphate buffer (pH 6.8) (mean ± s.d., n = 3). (**A**) Effect of PVP-K30 on drug dissolution; (**B**) Effect of P407 on drug dissolution.

Seven SDs with varying drug–polymer ratios were prepared (Table 1). As shown in Figure 2A, increasing the proportion of PVP-K30 while keeping P407 constant resulted in enhanced drug release compared with pure penfluridol, with PF-SD2 exhibiting the highest drug release within 1 h (approximately 98%). Interestingly, there was some difference in the initial dissolution rate according to the proportion of PVP-K30. PF-SD2 dissolved faster than PF-SD1 without PVP-K30, while the dissolution rate decreased with further increase in PVP-K30 proportion. This result may be explained by the concentration-dependent behavior of PVP-K30. At low concentrations, PVP-K30 improves wetting and dispersibility of the drug, resulting in increased dissolution [29,30]. Consequently, PF-SD2 dissolved faster than PF-SD1. However, high amounts of PVP-K30 form a high-viscosity gel layer upon hydration, which increases resistance to drug diffusion into the bulk medium, leading to slow drug dissolution [29,30]. This phenomenon explains the slower dissolution rate observed at higher PVP-K30 ratios. Accordingly, PF-SD2 likely represents the optimal proportion of PVP-K30 where the solubilizing effects of PVP-K30 are maximized before the onset of diffusion-limiting behavior.

**Figure 3:**
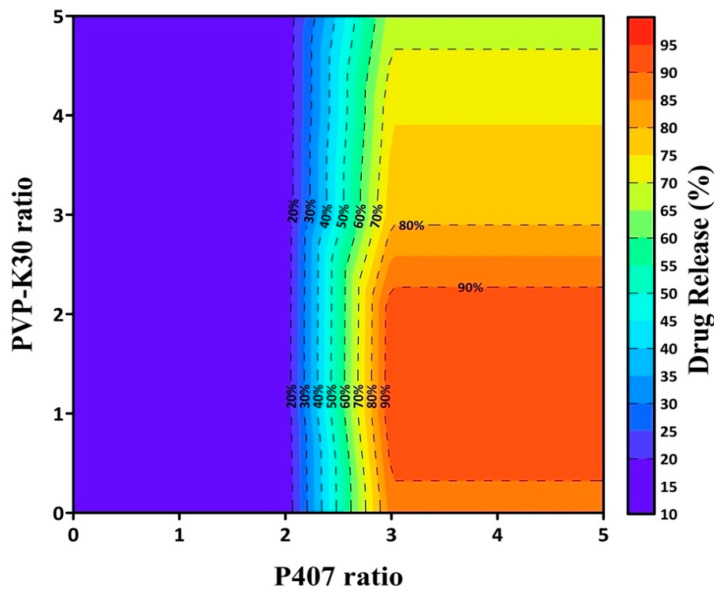
Contour plot of RFR-predicted drug release at 1 h as a function of PVP-K30 and P407 ratios.

To identify the optimal carrier ratio, dissolution data from the initial formulations (PF-SD1–PF-SD4) were analyzed using RFR. This computational method was used to predict the nonlinear relationship between carrier composition and the percentage of drug release at 1 h. The model predicted that a ratio of drug:P407:PVP-K30 = 1:3:1 would likely achieve > 85% drug release within 1 h (Figure 3). Based on this prediction, a second set of formulations (PF-SD5–PF-SD7) with varying ratios of P407 was prepared. Dissolution studies confirmed the model predictions: PF-SD5 (drug:P407:PVP-K30 = 1:3:1) achieved > 85% drug release within 1 h (Figure 2B). The RFR analysis indicates that drug release reached its maximum at a drug:P407:PVP-K30 ratio of 1:3:1, and further increases in the amounts of hydrophilic polymers did not enhance drug release (Figure 3). Consistently, Figure 2B reveals that PF-SD2 and PF-SD5 exhibit comparable dissolution behavior, achieving rapid and nearly complete drug dissolution within 1 h. These findings demonstrate the potential of the RFR method to accurately predict the optimal ratios of polymeric carriers and minimize the need for extensive experimental trials. Accordingly, PF-SD5 was selected as an optimal SD for further characterization.

### 3.3. In Vitro Characterization of PF-SD5

#### 3.3.1. Dissolution Behavior

The optimized PF-SD5 was evaluated for solubility and dissolution performance in comparison with PM and the pure penfluridol. As shown in Table 2, PF-SD5 demonstrated a 117-fold increase in solubility compared with the pure drug. Statistical analysis confirmed that the solubility of PF-SD5 was significantly higher than both PM and the pure drug.

**Figure 4:**
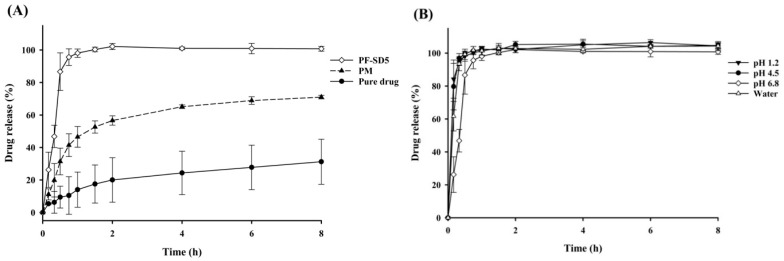
In vitro dissolution profiles (mean ± s.d., n = 3). (**A**) Dissolution profiles of pure drug, PM, and PF-SD5 in phosphate buffer (pH 6.8); (**B**) Dissolution profiles of PF-SD5 at different pH values.

Similar trends were observed in dissolution studies: PF-SD5 exhibited markedly enhanced drug release compared with the pure drug and the corresponding PM (Figure 4A). To further assess the robustness of the optimized formulation, the dissolution behavior of PF-SD5 was evaluated in dissolution media of varying pHs to determine its potential pH dependency. As shown in Figure 4B, dissolution was consistent from acidic to neutral pH, indicating that the drug release from PF-SD5 was pH-independent. The rapid and extensive drug dissolution from PF-SD5 was also confirmed in simulated gastric fluid (SGF) and simulated intestinal fluid (SIF) (Figure S1). Collectively, these findings suggest that PF-SD5 maintains rapid and extensive drug dissolution throughout the pH range of the gastrointestinal tract.

#### 3.3.2. Structural Characterization of PF-SD5

**Figure 5:**
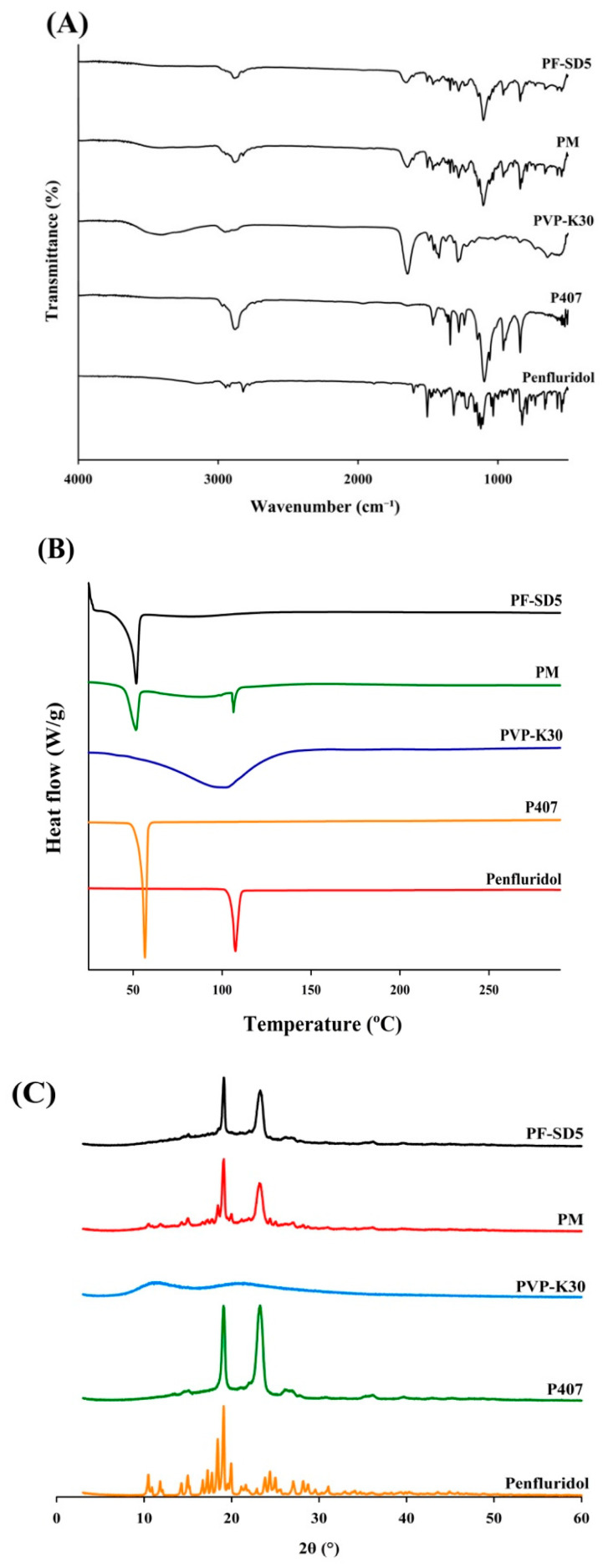
Structural analysis of PF-SD5. (**A**) FT-IR spectra, (**B**) DSC thermograms, (**C**) X-ray diffraction (XRD) diffractograms.

The FTIR spectrum of the pure penfluridol exhibited distinct absorption bands in the 1400–1600 cm^−1^ region, corresponding to aromatic C=C stretching vibrations within benzene rings (Figure 5A). The pronounced band at 3148 cm^−1^ is attributed to aromatic C–H stretching. Additionally, distinct aliphatic C–H stretches were evident near 2900 cm^−1^. The fingerprint region revealed multiple strong peaks align with extensive halogen substitution, notably C–Cl stretching between 600 and 800 cm^−1^, and C–F stretching bands in the 1157–1283 cm^−1^ range. These sharp and well-resolved signals are consistent with the crystalline nature of the unformulated drug substance. P407 exhibited characteristic C–O stretching at 1099 cm^−1^, and C–H stretching at 2880 cm^−1^. PVP-K30 showed a broad O–H stretching band centered at approximately 3411 cm^−1^, C–H stretching at 2948 cm^−1^, and a pronounced absorption at 1645 cm^−1^ corresponding to C=O carbonyl group stretching. Both carriers displayed unique fingerprint regions below 1500 cm^−1^, representing vibrations of the polymer backbone. The PM exhibited a combined spectral profile encompassing the major absorption bands of the drug and both carriers (Figure 5A). The absence of substantial shifts or disappearance of principal drug signals indicates that no chemical interactions occurred during simple mixing. In contrast, the SD formulation (PF-SD5) exhibited spectral changes compared with the spectra of pure penfluridol and individual carriers. For example, the hydroxyl group of penfluridol can form hydrogen bonds with the carbonyl group of PVP-K30, decreasing band intensity and a shift in C=O stretching of PVP-K30. The spectral alterations suggest a high degree of miscibility and successful formation of an SD system, reflected by drug–polymer compatibility.

The DSC thermograms exhibited distinct thermal transitions, indicating drug–polymer interactions and changes in crystallinity (Figure 5B). The pure drug exhibited a sharp endothermic peak at 107.47 °C, consistent with its melting point and crystalline nature. P407 showed a characteristic melting peak at 55.48 °C, whereas PVP-K30 displayed a broad endothermic peak at 100.16 °C, typical of its amorphous structure and hygroscopic properties [31,32]. The PM exhibited multiple endothermic events at 51.01 °C, 84.06 °C, and 107.13 °C, reflecting the combined transitions of the drug and polymers, which indicated limited interactions between components and the retention of crystalline drug domains. In contrast, PF-SD5 exhibited shifted endothermic peaks at 49.94 °C and 81.50 °C, accompanied by the disappearance of the original melting peak of the drug. This observation strongly suggests that penfluridol was converted to an amorphous form within the polymer matrix or dissolved in the molten carrier before reaching the fusion temperature [33].

To clarify changes in drug crystallinity, PF-SD5 was subjected to XRD analysis. As shown in Figure 5C, the XRD pattern of the pure drug showed distinct sharp peaks at 10.49°, 14.99°, 17.25°, 17.75°, 18.41°, 19.09°, 19.95°, and 24.39°, confirming its crystalline nature. The diffraction pattern of P407 exhibited characteristic peaks at approximately 19.07° and 23.25°, indicative of its semi-crystalline nature, whereas PVP-K30 showed a broad, diffuse halo, characteristic of an amorphous polymer, confirming its lack of crystallinity [34]. The PM presented characteristic peaks corresponding to both the drug and P407, indicating that the crystalline structures of these components were retained upon simple physical mixing, with no alteration in drug crystallinity. In contrast, PF-SD5 displayed two prominent peaks at 19.11° and 23.27°, which closely matched the characteristic peaks of P407, while all crystalline peaks attributable to penfluridol disappeared. This finding suggests that the drug exists in an amorphous form, which may contribute to its enhanced solubility and bioavailability [35].

#### 3.3.3. Morphological Characterization of PF-SD5

**Figure 6:**
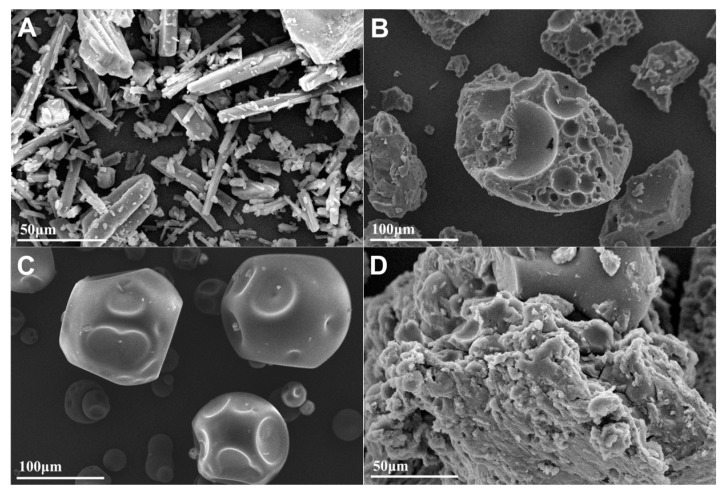
SEM images of (**A**) pure penfluridol at 50 μm scale, (**B**) P407 at 100 μm scale, (**C**) PVP-K30 at 100 μm scale, (**D**) PF-SD5 at 50 μm scale.

The surface morphology of pure penfluridol, carrier polymers, and PF-SD5 was examined using scanning electron microscopy (SEM), as shown in Figure 6A–D. The SEM micrograph of the pure drug revealed distinct, well-defined crystalline structures with sharp edges, indicating the crystalline nature of penfluridol (Figure 6A). In contrast, PF-SD5 displayed a uniform, smooth, and nearly featureless surface without visible crystalline particles, confirming that the drug has been molecularly dispersed within the polymeric matrix in an amorphous form (Figure 6D). These morphological findings are consistent with the DSC and XRD results (Figure 5).

### 3.4. Pharmacokinetic Study in Rats

**Figure 7:**
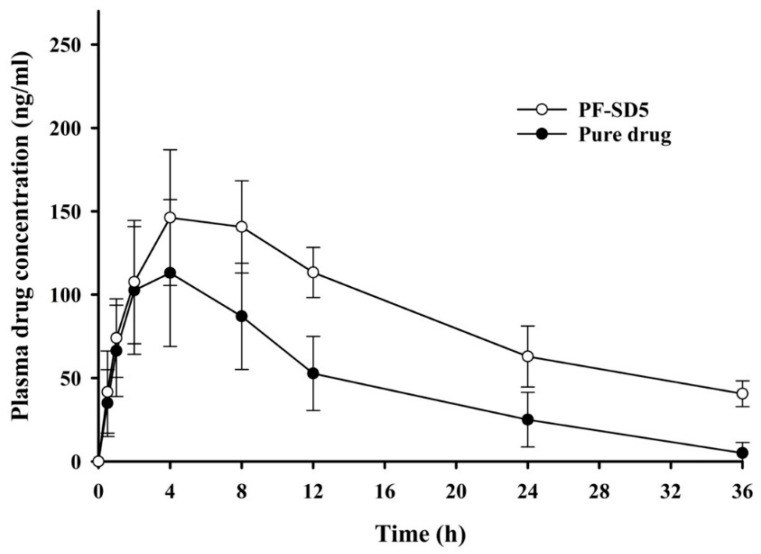
Pharmacokinetic profiles of penfluridol following oral administration of pure drug or PF-SD5 in rats. The dose was equivalent to 10 mg/kg of penfluridol (mean ± s.d., n = 6).

The pharmacokinetic parameters of penfluridol following oral administration of the pure drug or PF-SD5 in rats are presented in Table 3 and Figure 7. The area under the plasma concentration-time curve (AUC) of PF-SD5 (3144 ± 429 ng·h/mL) was significantly higher than that of the pure drug (1661 ± 610 ng·h/mL), indicating enhanced systemic exposure with approximately 189% relative bioavailability. This improvement can be attributed to increased drug solubility through amorphization and enhanced wetting by the hydrophilic polymeric matrix, which facilitated drug dissolution and absorption in the gastrointestinal tract. Although the C_max_ value increased from 115 ± 45.4 ng/mL for pure penfluridol to 154 ± 38.2 ng/mL for PF-SD5, this difference was not statistically significant. This finding suggests that PF-SD5 increased systemic drug exposure without causing an extreme spike in plasma peak concentration. Clinically, this finding could be advantageous for mitigating side effects associated with antipsychotics, such as extrapyramidal symptoms, weight gain, and hyperprolactinemia, which are dose- and concentration-dependent [36]. Maintaining comparable or non-significantly increased peak plasma concentrations while enhancing systemic exposure may improve the pharmacokinetic profile and tolerability of penfluridol. Formulations of antipsychotic drugs that minimize excessive peak concentrations while sustaining systemic exposure are generally preferred to improve patient tolerability compared with conventional immediate-release formulations [37].

Overall, these findings clearly demonstrate that the enhanced dissolution profile of the PF-SD5 formulation effectively resulted in a significant improvement in the oral bioavailability of penfluridol. Notably, unlike other penfluridol formulations reported in the literature, which often lack in vivo bioavailability data, this study provides strong evidence linking in vitro dissolution enhancement with in vivo pharmacokinetic performance. The consistency between dissolution and bioavailability results supports the reliability of SD technology as a predictive and effective formulation strategy. The ternary SD system, incorporating P407 and PVP-K30, provides a synergistic combination that successfully overcomes multiple barriers to the oral absorption of this poorly soluble drug.

## 4. Conclusions

The SD formulation (PF-SD5) achieved a marked increase in the aqueous solubility, dissolution rate, and oral bioavailability of penfluridol. Structural and morphological analyses confirmed the conversion of penfluridol to an amorphous state, which contributed to improved dissolution and oral absorption. Furthermore, the application of RFR modeling provided valuable insights into the influence of formulation components on drug release, thereby reducing the need for extensive experimental evaluations. Collectively, these findings demonstrate that combining SD technology with computational prediction offers an effective strategy to overcome the solubility and dissolution limitations of penfluridol.

## Figures and Tables

**Table 1:** Components and ratios of SDs.

Formulation	Ratio (*w*/*w*/*w*)		
	Penfluridol	P407	PVP-K30
PF-SD1	1	5	0
PF-SD2	1	5	1
PF-SD3	1	5	3
PF-SD4	1	5	5
PF-SD5	1	3	1
PF-SD6	1	2	1
PF-SD7	1	1	1

**Table 2:** Aqueous solubility of penfluridol from different formulations (mean ± s.d., n = 3).

Formulation	Solubility (μg/mL)
Pure drug	0.78 ± 0.02
PM	54.6 ± 8.81 *
PF-SD5	91.2 ± 6.29 *

* *p* < 0.05, compared to pure drug.

**Table 3:** Pharmacokinetic parameters of penfluridol following oral administration of pure drug or PF-SD5 in rats. The dose was equivalent to 10 mg/kg of penfluridol (mean ± s.d., n = 6).

Parameters	Pure Drug	PF-SD5
C_max_ (ng/mL)	115 ± 45.4	154 ± 38.2
T_max_ (h)	4.3 ± 2.0	6.0 ± 2.2
AUC (ng × h/mL)	1661 ± 610	3144 ± 429 *

* *p* < 0.05, compared to pure drug.

## Data Availability

The original contributions presented in this study are included in the article/Supplementary Material. Further inquiries can be directed to the corresponding author.

## Supplementary Materials

The following supporting information can be downloaded at: https://www.mdpi.com/article/10.3390/pharmaceutics17121546/s1, Figure S1: Dissolution profiles of pure drug and PF-SD5 in SGF and SIF.
