# Supplementary material for: Combined Experimental and Computational Approaches for Ternary Solid Dispersions to Enhance the Oral Bioavailability of Penfluridol
Source: Pharmaceutics. 2025 Nov 30;17(12):1546. doi: 10.3390/pharmaceutics17121546 (PMC12736625; doi:10.3390/pharmaceutics17121546)
Supplement: Supplementary file 1 [file pharmaceutics-17-01546-s001.zip › pharmaceutics-4009979-supplementary.pdf]

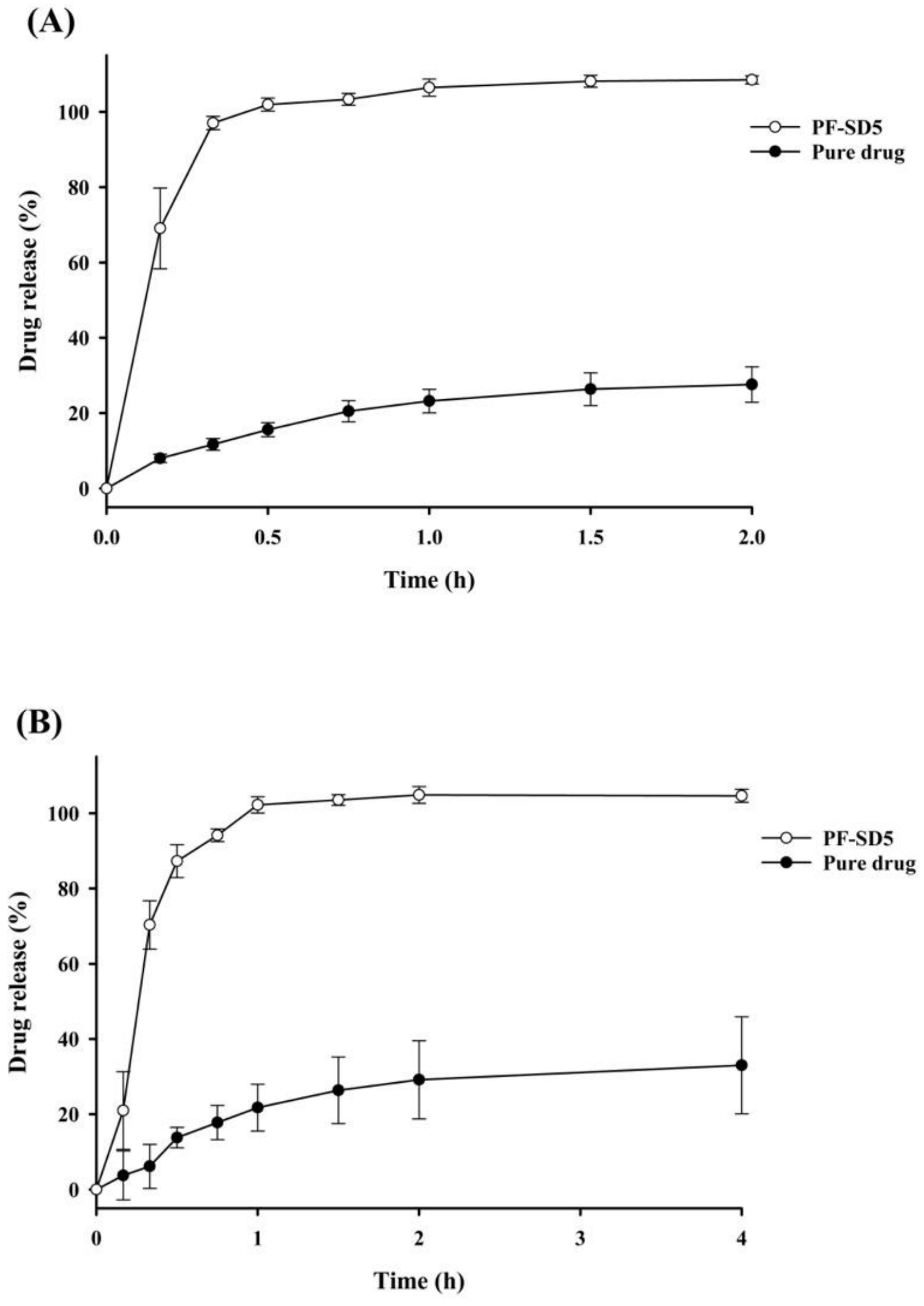

Figure S1. Dissolution profiles of pure drug and PF-SD5 in (A) simulated gastric fluid (SGF) and (B) simulated intestinal fluid (SIF) (mean ± s.d., n = 3).
